# Three-Plug Technique for Mitral Paravalvular Leak Closure in Marfan Syndrome

**DOI:** 10.7759/cureus.77920

**Published:** 2025-01-24

**Authors:** Vlasis Ninios, Georgios E Papadopoulos, Andreas Ioannides, Sotirios Evangelou, Ilias Ninios

**Affiliations:** 1 2nd Cardiology Department, European Interbalkan Medical Center, Thessaloniki, GRC; 2 1st Cardiology Department, University Hospital of Ioannina, Ioannina, GRC

**Keywords:** amplatzer plug device, marfan's syndrome, mitral valve disease, mitral valve surgery, paravalvular leak closure

## Abstract

Paravalvular leaks (PVLs) represent a challenging complication following surgical mitral valve replacement, particularly in patients with connective tissue disorders such as Marfan syndrome. This report presents the case of a 32-year-old female with Marfan syndrome who presented with progressive dyspnea and New York Heart Association (NYHA) functional class II-III symptoms 10 years post-mitral valve replacement for severe mitral regurgitation. Transthoracic and transesophageal echocardiography identified an 8 × 9 mm paravalvular defect with compliant borders, deemed suitable for percutaneous closure. Surgical re-intervention was considered high risk by the Heart Team, leading to the decision to proceed with a minimally invasive approach.

Under general anesthesia, the defect was crossed using a steerable guide catheter and stabilized with a super-stiff wire. A stepwise closure strategy employing three Amplatzer Vascular Plug III devices (two 14 × 5 mm and one 10 × 5 mm) successfully reduced the regurgitation to trace levels. The patient experienced significant symptomatic improvement, achieving NYHA functional class I at six months post-procedure, with echocardiography confirming the durable closure of the defect.

This case highlights the technical nuances and innovative use of multiple devices for mitral PVL closure, particularly in the context of connective tissue disorders. Multimodality imaging, strategic device selection, and operator expertise are critical for optimizing outcomes in such high-risk scenarios.

## Introduction

Paravalvular leak (PVL) is a common complication following valve replacement surgery, caused by incomplete sealing of the prosthetic sewing ring to the native tissue [1], which can occur due to several factors, including infection (endocarditis), tissue degeneration, calcification, and technical issues during the initial surgery, such as suboptimal positioning or suture tension [2]. In connective tissue disorders such as Marfan syndrome, structural abnormalities of the mitral valve and annulus increase the risk of complications such as mitral valve prolapse, regurgitation, and PVLs following prosthetic valve implantation [3]. Small PVLs are detected in up to 50% of patients following valve replacement surgery, most of whom remain asymptomatic [4]. However, the prevalence varies depending on the valve type, with higher detection rates in mechanical valves compared to bioprosthetic valves due to their rigid structure and complex annular dynamics. In 1-5% of cases, PVLs become clinically significant, presenting with symptoms such as heart failure and hemolysis [5]. These challenges are amplified in patients with connective tissue disorders such as Marfan syndrome, where native tissue fragility complicates management and increases the likelihood of adverse outcomes.

The traditional treatment for symptomatic PVLs is surgical reoperation, which remains the definitive approach. However, surgical reintervention is associated with significant risks, with mortality rates reported as high as 22% [6,7]. For high-risk patients, transcatheter PVL closure has emerged as a viable alternative. First reported in 2003 using a ductal coil [8], this minimally invasive technique has been utilized for over two decades. Despite its potential, its application remains limited to specialized centers due to procedural complexity, a steep learning curve requiring advanced operator expertise, and the limited availability of devices specifically designed for PVL closure. Outcomes are largely based on case studies and small series, with variability in results linked to differences in device selection and procedural methods.

In this report, we present the case of a young female with Marfan syndrome who underwent successful transcatheter closure of a symptomatic mitral PVL using an innovative three-plug technique. This case highlights the technical challenges and tailored solutions required to manage complex PVLs in high-risk patients.

## Case presentation

A 32-year-old female with a known history of Marfan syndrome presented with progressive dyspnea on exertion over two years, with a marked worsening in symptoms during the three months prior to her presentation.

The patient had undergone surgical mitral valve replacement with a 29 mm St. Jude Medical™ bileaflet mechanical valve 10 years earlier due to severe mitral regurgitation caused by Barlow’s disease, a condition characterized by myxomatous degeneration of the mitral valve, leading to prolapse and regurgitation. The postoperative course was uneventful, and she was maintained on oral anticoagulation therapy with warfarin. At presentation, she was classified as New York Heart Association (NYHA) functional class II-III, having recently worsened from class II over the prior three months.

**Figure 1:**
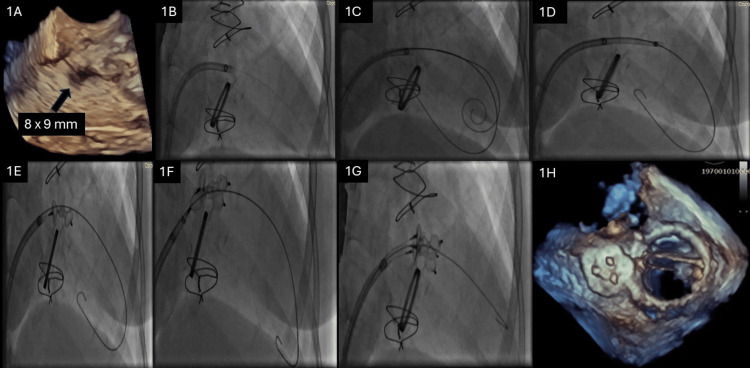
Three-plug technique for mitral paravalvular leak closure in Marfan syndrome (A) Pre-procedural 3D echocardiography with 8 x 9 mm defect. (B) Defect crossed with 0.014-inch wire through 15F SGC. (C) Super stiff wire and 0.035-inch buddy-safety wire through the defect. (D) A 7F guide sheath insertion. (E) First AVP III 14 x 5 mm deployment. (F) Second AVP III 14 x 5 mm deployment. (G) Third AVP III 10 x 5 mm deployment. (H) Final result. 3D, three-dimensional; SGC, steerable guide catheter; AVP, Amplatzer Vascular Plug

Echocardiographic evaluation, including transthoracic and transesophageal imaging, revealed a round-shaped paravalvular defect measuring 8 × 9 mm, located in the anterolateral region of the mitral valve annulus relatively remote from the valve ring (Figure 1A). The defect had compliant borders, suggesting suitability for percutaneous closure. Pulmonary artery systolic pressure was mildly elevated at 40 mmHg, and left ventricular function was preserved.

Given the patient’s history of Marfan syndrome and previous surgical mitral valve replacement, in addition to the favorable anatomy of the defect, including its compliant borders and remote location, surgical re-intervention was deemed high risk, and the Heart Team decided to proceed with percutaneous closure.

The procedure was performed under general anesthesia with fluoroscopic and echocardiographic guidance. A steerable 15F guide catheter was advanced toward the defect, which was successfully crossed using a 0.014-inch wire (Figure 1B). This wire was exchanged for a Safari™ pre-shaped guidewire (Boston Scientific, Marlborough, MA, USA) to provide stability for device delivery and a 0.035-inch buddy-safety wire to enhance catheter stability and reduce the risk of device malposition during delivery. (Figure 1C). A 7F delivery sheath was then inserted into the defect (Figure 1D).

The first attempt at closure involved deploying an Amplatzer Vascular Plug III (AVP III) device measuring 14 × 5 mm (Figure 1E). While this achieved partial closure, a residual 7 mm gap remained, likely due to the defect’s irregular shape and compliant borders. A second AVP III device of the same size was deployed adjacent to the first device, which reduced the gap but left mild residual regurgitation (Figure 1F). Finally, a third AVP III device measuring 10 × 5 mm was deployed (Figure 1G). This achieved significant improvement in mitral regurgitation, with only trace residual regurgitation noted on immediate post-procedural echocardiography (vena contracta width of 3 mm, an effective regurgitant orifice area of approximately 0.05 cm², and a regurgitant volume of 10 mL). Pulmonary artery systolic pressure decreased to 35 mmHg. All three devices were deployed simultaneously to ensure a stable and complete seal (Figure 1H).

The patient tolerated the procedure well and was discharged in stable condition. She continued anticoagulation therapy with warfarin. Scheduled follow-ups at 3, 6, and 12 months included clinical assessments and transthoracic echocardiography to monitor device stability, residual regurgitation, and thrombus formation. At six months, the patient remained asymptomatic (NYHA class I), with durable closure confirmed on echocardiography.

## Discussion

PVLs remain a challenging complication of prosthetic valve replacement, particularly in patients requiring repeated interventions. While the incidence of clinically significant PVLs is relatively low at 1-5%, it escalates dramatically to 25-40% in cases of reoperations of both mitral and aortic valves [4,9]. Heart failure, a common clinical manifestation, results from volume overload due to regurgitant flow through the PVL. This can lead to progressive left ventricular dysfunction, frequent hospitalizations, and reduced exercise capacity, all of which significantly diminish quality of life and increase long-term mortality. Hemolysis, another hallmark of PVLs, occurs due to mechanical destruction of red blood cells caused by high-velocity turbulent jets passing through the defect. Addressing these complications through effective PVL management is critical to improving prognosis and quality of life.

The procedure benefited significantly from real-time guidance provided by three-dimensional transesophageal echocardiography (3D-TEE), which was instrumental in navigating the defect, selecting the appropriate device sizes, and ensuring accurate deployment. For example, 3D-TEE allowed for precise crossing of the defect with the guidewire and confirmed device placement without impinging on the function of the prosthetic valve leaflets.

Mitral PVL closure, in particular, involves unique technical challenges compared to PVLs in other valve positions. The complex anatomy of the mitral annulus and its proximity to the prosthetic valve leaflets require precise navigation to avoid interference with valve function. Additionally, achieving hemostasis with multiple devices can be challenging, especially in defects with irregular or compliant borders. These challenges underscore the need for meticulous pre-procedural planning, advanced imaging techniques, and operator expertise to mitigate risks such as device embolization or valve dysfunction.

Currently, there are no devices specifically designed for PVL closure, which presents challenges in achieving optimal outcomes. Devices such as the AVP III, originally designed for vascular occlusion, have been adapted for this purpose due to their flexibility and ability to conform to irregular defect shapes. However, ongoing research into specialized devices tailored to PVL closure is promising. These advancements aim to address the unique challenges posed by PVLs, such as irregular defect morphology and close proximity to prosthetic valve structures, and may further improve procedural success rates and reduce complications.

This case contributes to the growing body of evidence supporting the use of transcatheter techniques for PVL management, particularly in high-risk patients with connective tissue disorders such as Marfan syndrome. The approach demonstrated here emphasizes the role of advanced imaging, strategic device selection, and stepwise closure strategies in achieving optimal outcomes. Furthermore, it underscores the need for future research to develop devices specifically designed for PVL closure, standardize procedural techniques, and explore long-term outcomes in diverse patient populations. These efforts will be critical to advancing the field and improving the prognosis for patients with complex PVLs.

## Conclusions

This case demonstrates the feasibility and durability of a three-plug transcatheter technique for mitral PVL closure in a high-risk patient with Marfan syndrome, achieving long-term defect closure and symptom resolution. The symptoms of hemolysis and congestive heart failure, hallmark manifestations of PVLs, underscore the clinical significance of timely and effective intervention. The use of advanced imaging modalities, particularly 3D-TEE, was critical in guiding the procedure, choosing the appropriate device, and ensuring precise device placement. The technique's adaptability to irregular defect anatomy suggests its potential applicability to other anatomically complex PVLs, especially in patients with prior surgical histories or significant comorbidities. Future advancements in dedicated PVL closure devices, imaging technologies, and procedural techniques will further enhance outcomes and broaden the clinical utility of transcatheter therapies in managing challenging PVLs.
